# Comparative Meta-Analysis of Chemical and Biological Strategies for the Management of Wheat Stripe Rust (*Puccinia striiformis* f. sp. *tritici*) Under Global Agro-Ecological Conditions

**DOI:** 10.3390/plants15030412

**Published:** 2026-01-29

**Authors:** Ilham Dehbi, Salah-Eddine Laasli, Mouna Janati, Khadija Benamar, Moussa El Jarroudi, Hamid Mazouz, Rachid Lahlali

**Affiliations:** 1 Phytopathology Unit, Department of Plant and Environment Protection, Ecole Nationale d’Agriculture de Meknes, km. 10, Route Haj Kaddour, Meknes BP S/40, Morocco; il.dehbi@edu.umi.ac.ma (I.D.);; 2 Laboratory of Biotechnology and Bioresource Valorization, Moulay Ismail University of Meknes, Zitoune, Meknes BP 11201, Morocco; m.janati@edu.umi.ac.ma (M.J.);; 3 Crop Protection Unit, Agronomic and Veterinary Institute (IAV), Rabat BP 6202, Morocco; 4 Laboratory of Microbial Biotechnology and Bioactive Molecules, Faculty of Sciences and Technologies, Sidi Mohamed Ben Abdellah University, Imouzzer Road, Fez BP 2202, Morocco; khadija.benamar2@usmba.ac.ma; 5 SPHERES Research Unit, Department of Environmental Sciences and Management, University of Liège, 6700 Arlon, Belgium

**Keywords:** wheat stripe rust, meta-analysis, fungicides, biocontrol agents, yield response, agroecological disease management

## Abstract

Wheat stripe rust, caused by *Puccinia striiformis* f. sp. *tritici*, threatens global wheat production, with climate change intensifying its spread. This meta-analysis, following PRISMA protocol, evaluated chemical and biological control methods through a systematic review of literature (2005–2025), identifying 12 peer-reviewed studies with 156 experimental comparisons under various conditions. Random effects models assessed treatment impacts on disease severity and grain productivity using standardized mean differences (SMDs). Chemical control significantly reduced stripe rust severity (SMD = −1.04) and improved productivity (SMD = 1.30), with low to moderate variability and consistent yield responses. Effectiveness varied by active ingredients and wheat types, with the greatest benefits in highly susceptible varieties. Biological control agents, particularly *Bacillus*, *Pseudomonas*, and *Trichoderma* species, also reduced disease severity (SMD = −2.19) and increased yield (SMD = 2.39), though with greater heterogeneity reflecting strain-specific and environmental effects. Chemical fungicides provided more predictable disease control, while biological agents offered significant yield increases with agroecological benefits. This meta-analysis demonstrates complementary roles for both approaches, strongly supporting integrated disease management combining plant resistance, optimal fungicide use, and strategic biological control to enhance resilience and sustainability of global cereal production systems.

## 1. Introduction

Global cereal production, particularly wheat (*Triticum* spp.), is a fundamental pillar of food security [1]. With more than 217 million hectares cultivated worldwide, wheat is the main source of calories and protein for more than a third of the world’s population (FAO, 2023) [2,3,4]. In Morocco, this crop occupies a strategic place in the agricultural system, covering 62% of cultivated land, accounting for approximately 55% of the total value added by crop production and making a major contribution to rural income and socio-economic stability (ONSSA, 2022) [4,5]. However, the sustainability of this production is continually threatened by several biotic constraints, including stripe rust, caused by the fungus *Puccinia striiformis* f. sp. *tritici* (Pst), which is among the most devastating.

Stripe rust is characterized by yellow-orange pustules arranged in parallel bands on the leaves, causing a significant reduction in photosynthesis and, consequently, yield [6,7,8]. Under favorable climatic conditions—cool temperatures (10–15 °C) and high humidity—epidemics can spread rapidly, resulting in yield losses of up to 70–100% in severe cases [9]. In the context of climate change, the dynamics of the pathogen have changed profoundly: the emergence of more aggressive strains adapted to higher temperatures has expanded the geographical area of infection, including traditionally unaffected semi-arid areas [10,11]. These changes reinforce the need for integrated, sustainable management strategies adapted to new epidemic pressures.

Historically, chemical control through the application of synthetic fungicides, particularly triazoles (such as tebuconazole or propiconazole) and strobilurins (such as azoxystrobin or bixafen), has been the most widely used method for controlling stripe rust. The results compiled in this database show that these products reduce disease severity by an average of 70 to 85%, confirming their agronomic effectiveness [12,13,14,15]. However, this growing dependence on chemicals raises several concerns: the emergence of resistant strains of the pathogen, documented in several regions of the world; the environmental impact linked to soil and water contamination; health risks for users and consumers; and the decline of beneficial microbial biodiversity in agroecosystems [16,17]. Thus, although fungicides remain indispensable tools in the management of acute epidemics, their exclusive use is scientifically considered unsustainable in the long term.

Given these limitations, biocontrol strategies are attracting growing interest. These strategies rely on the use of beneficial microorganisms, mainly bacteria of the genus *Bacillus* or *Pseudomonas*, and fungi such as *Trichoderma*—capable of reducing pathogenic infection through various mechanisms: direct inhibition of pathogen growth through the production of antifungal metabolites, competition for nutrients and infection sites, and induction of systemic resistance in the host plant (ISR, SAR) [18,19,20,21,22,23,24]. The studies included in this meta-analysis [18,19] report that applications of *Bacillus subtilis* can reduce the severity of stripe rust by 40 to 60%, while improving wheat vigor and yield. In addition, these treatments have the advantage of being compatible with agroecological approaches, leaving no chemical residues and contributing to soil health [25,26].

However, their effectiveness remains influenced by environmental factors (temperature, humidity, cultivar type, etc.) and technical factors (application method, concentration, plant stage, etc.), requiring a comprehensive quantitative assessment to identify robust and generalizable trends. In a context where numerous but heterogeneous studies are available, a meta-analysis allows the results from various independent experiments to be statistically integrated in order to obtain an overall quantitative estimate of the effectiveness of different control strategies in plant pathology [27,28]. For instance, in wheat disease research, meta-analytical frameworks have been applied to evaluate management strategies for 61 cultivar mixtures on wheat stripe rust in 11 reference studies using the random effect model and the fixed effect model with an effect size ranged from 22.5% to 33.4% with an average of 28.0% to contrasting resistance backgrounds, demonstrating the ability of this approach, quantify disease suppression and identify key factors influencing control efficacy across multiple environments [29].

This approach aims to answer three major scientific questions: (i) what is the average effectiveness of chemical and biological treatments on wheat severity and yield; (ii) what factors moderate these effects (type of agent, stage of application, experimental conditions, cultivar); (iii) and to what extent can biocontrol be a reliable or complementary alternative to conventional fungicides.

This meta-analysis is based on a corpus of more than 60 experimental trials divided between chemical and biological treatments, covering a wide variety of agroecological contexts. The objective is to provide an integrated, scientific, and objective view of the performance of both strategies against wheat stripe rust and to identify generalizable trends across agroecological contexts, with a view to transitioning to more resilient and sustainable cereal production systems at a global scale.

## 2. Results

### 2.1. Chemical Control

#### 2.1.1. Effect of Chemical Control on Stripe Rust Disease Severity and Yield

**Figure 1:**
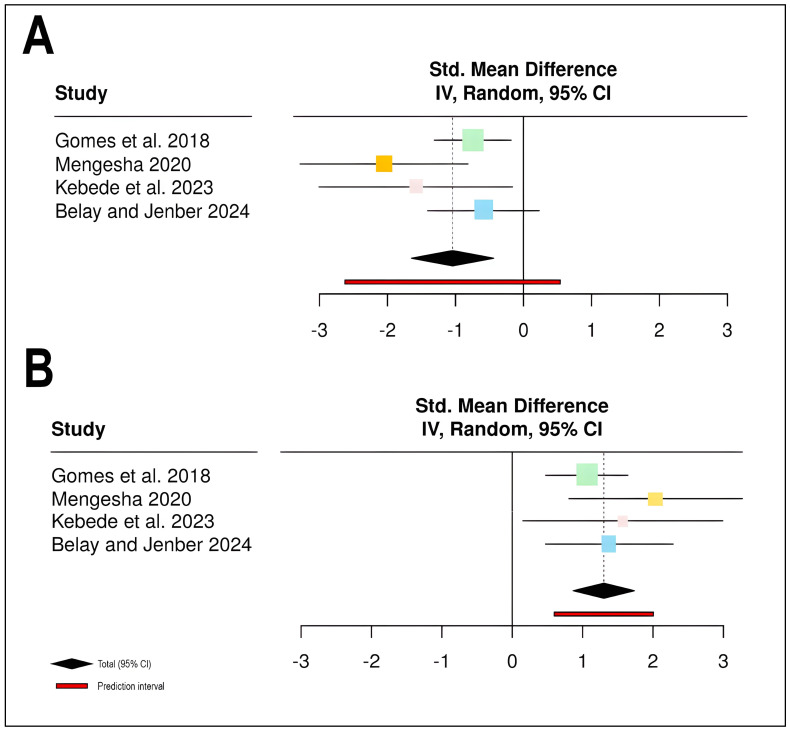
Meta-analysis forest plots demonstrating the effectiveness of chemical control interventions on stripe rust disease management and wheat productivity: (**A**) Standardized mean difference (SMD) for stripe rust disease severity across four studies (*n* = 4) [12,13,14,15], showing disease severity reduction (negative SMD values indicate disease reduction). (**B**) Standardized mean difference for wheat yield across the same four studies, demonstrating yield increase (positive SMD values indicate yield improvement). Effect sizes were pooled using a random-effects model with inverse variance (IV) weighting. Each square represents the point estimate of individual study effects, with the square size proportional to study weight in the meta-analysis. Horizontal lines represent 95% confidence intervals (CI) for each study. The black diamond at the bottom indicates the pooled effect estimate with its 95% CI, while the red bar represents the prediction interval for future studies. The vertical dashed line at zero represents no effect.

The meta-analysis of chemical control effects on stripe rust severity included four studies [12,13,14,15] with a total of 72 participants in treatment groups and 48 in control groups (Table 1; Figure 1). The pooled analysis revealed that chemical control significantly reduced disease severity compared to control treatments, with a standardized mean difference (SMD) of −1.04 (95% CI: −1.65 to −0.44, *Z* = −3.39, *p* = 0.0007). All four individual studies demonstrated negative effect sizes favoring the treatment group, with study-specific weights ranging from 13.9% to 40.1%. Heterogeneity assessment indicated moderate variability among studies (*I*^2^ = 40.6%, *τ*^2^ = 0.1530, *χ*^2^ = 5.05, *df* = 3, *p* = 0.1681), suggesting that approximately 40.6% of the observed variance reflects true differences in treatment effects rather than sampling error. The prediction interval [−2.63; 0.54] indicates that while most future studies would likely show beneficial effects, some variability in treatment response may be expected (Figure 1A).

For yield outcomes, the same four studies contributed data from identical sample sizes (72 treatment, 48 control participants). Chemical control demonstrated a substantial positive effect on yield, with a pooled SMD of 1.301 (95% CI: 0.867 to 1.735, *Z* = 5.88, *p* < 0.0001), indicating highly significant yield improvements in treated plots compared to controls. The analysis revealed no statistical heterogeneity among studies (*I*^2^ = 0.0%, *τ*^2^ = 0, *χ*^2^ = 2.21, *df* = 3, *p* = 0.5304), suggesting consistent treatment effects across different study contexts. All individual studies showed positive effect estimates, with study weights ranging from 9.3% to 55.3%, and confidence intervals for all studies excluded the null value of zero. The narrow prediction interval [0.597; 2.005] further confirms the consistency of positive yield responses to chemical control across studies. These findings collectively demonstrate that chemical fungicide applications effectively reduce stripe rust severity while simultaneously improving wheat yields, with particularly robust evidence for yield enhancement given the absence of between-study heterogeneity (Figure 1B).

#### 2.1.2. Publication Bias Assessment for Chemical Control Studies

**Figure 2:**
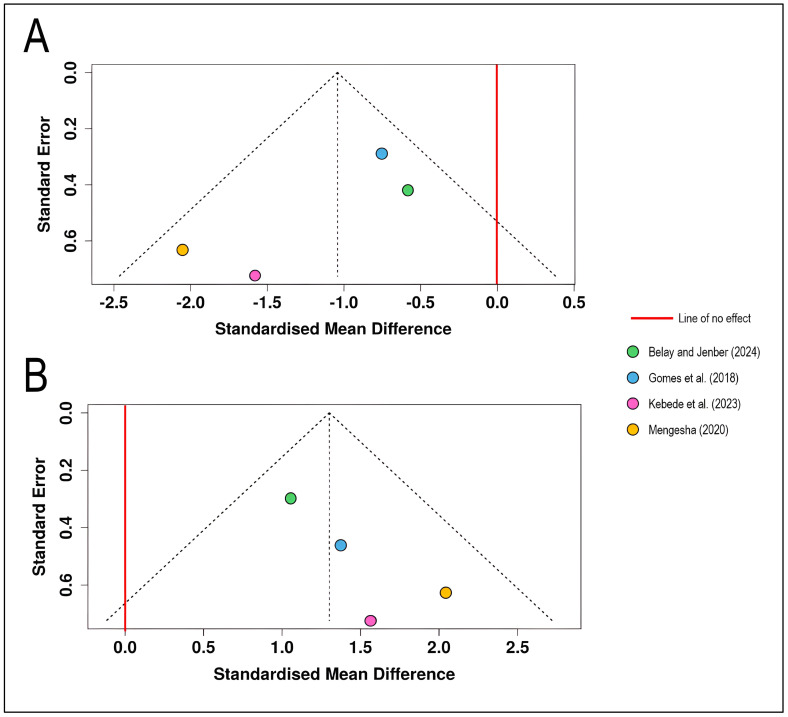
Funnel plots for visual assessment of publication bias in the meta-analysis of chemical control effects on wheat stripe rust: (**A**) Funnel plot for disease severity outcomes (*n* = 4 studies) showing the relationship between standardized mean difference (*x*-axis) and standard error (*y*-axis, inverted scale with larger, more precise studies at the top). (**B**) Funnel plot for wheat yield outcomes (*n* = 4 studies) [12,13,14,15]. Each colored circle represents an individual study, positioned according to its effect size and precision. The dashed diagonal lines form a pseudo-confidence region representing the expected 95% distribution of studies in the absence of heterogeneity and bias. The solid red vertical line indicates the line of no effect (SMD = 0).

The funnel plot for disease severity outcomes displays the standardized mean differences from four studies [12,13,14,15] plotted against their standard errors (Figure 2). All effect estimates lie to the left of the line of no effect (SMD = 0), indicating consistent disease severity reduction across studies. The distribution of studies shows relative symmetry around the pooled effect estimate, with studies scattered across different precision levels (standard errors ranging from approximately 0.3 to 0.7). The four studies fall largely within the pseudo-95% confidence limits defined by the funnel boundaries, suggesting no obvious asymmetry (the Egger’s test does not support the presence of funnel plot asymmetry (*intercept* = −2.59, 95% CI = −5.23–0.05, *t* = −1.922, *p* = 0.195). The relatively balanced distribution suggests minimal evidence of small-study effects or selective publication of statistically significant results. The funnel plot does not indicate a potential publication bias (Figure 2A).

The funnel plot for yield outcomes presents the same four studies with effect estimates positioned to the right of the line of no effect, reflecting positive yield responses to chemical control. The studies demonstrate moderately symmetrical distribution around the pooled effect, with standard errors ranging from approximately 0.3 to 0.7, similar to the severity analysis. All four studies fall within the expected funnel boundaries, and no obvious gaps or asymmetries are apparent that would suggest publication bias or selective reporting of positive results (*intercept* = 1.93, 95% CI = 0.53–3.34, *t* = 2.698, *p* = 0.114). The study by [12] shows the smallest standard error (highest precision), while the other three studies exhibit comparable precision levels (Figure 2B).

**Figure 3:**
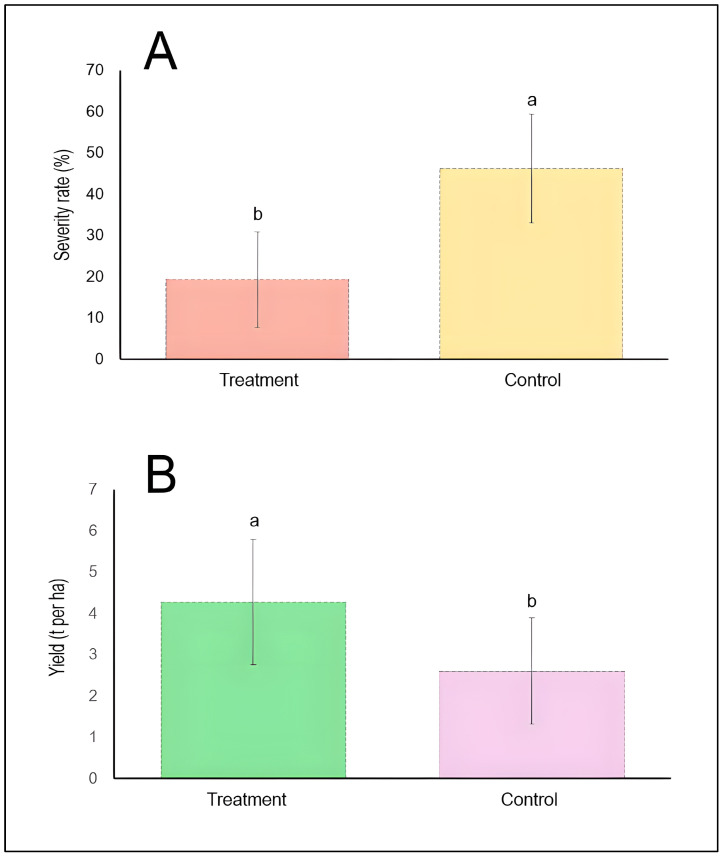
Comparative efficacy of chemical control on stripe rust severity (**A**) and wheat yield (**B**). Bars represent mean values pooled across all included studies (*n* = 4), with vertical error bars indicating standard deviation. Different lowercase letters (a, b) above bars denote statistically significant differences between treatment and control groups (*p* < 0.05) according to the Tukey HSD test. The dashed horizontal line at the top of each bar represents the upper limit of the standard deviation.

A general comparison between treatments and controls was conducted (Figure 3). The mean disease severity rates for chemically treated and untreated control plots were illustrated. The treatment group exhibited a mean severity of approximately 19%, while the control group showed substantially higher disease severity at approximately 46%. The ANOVA test confirms that chemical control treatments significantly reduced stripe rust severity by more than 50% relative to untreated controls (*p* < 0.05) (Figure 3A). The corresponding yield performance measured in metric tons per hectare for both treatment categories was also highlighted. Chemically treated plots produced a mean yield of approximately 4.3 t/ha, significantly exceeding the control group yield of approximately 2.6 t/ha. The treatment group achieved approximately 65% higher grain yield compared to untreated controls, demonstrating a strong positive relationship between disease suppression and crop performance (Figure 3B).

**Figure 4:**
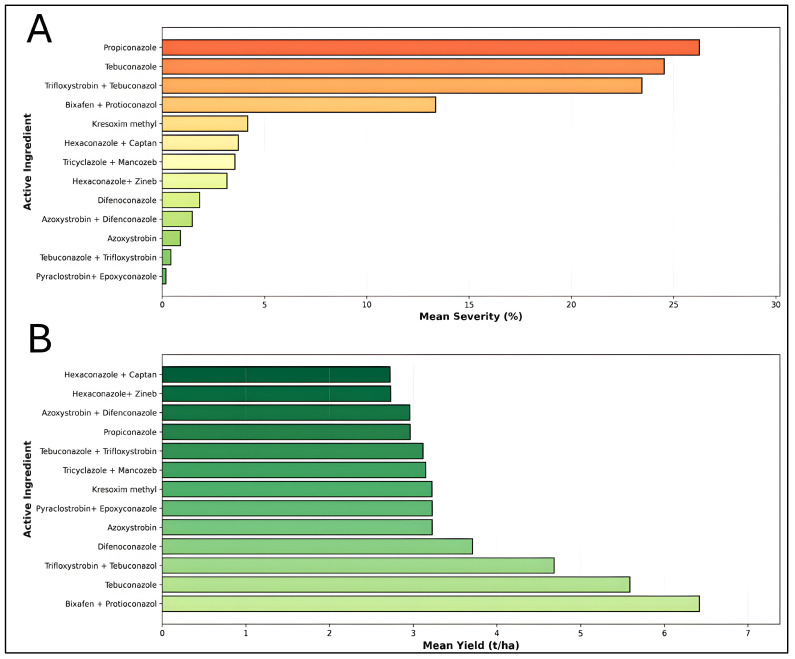
Comparative effectiveness of thirteen fungicide active ingredients and combinations in managing wheat stripe rust disease severity and enhancing grain yield: (**A**) Mean stripe rust disease severity (%) for each fungicide treatment, ranked from highest (poorest control) to lowest (best control) severity. (**B**) Mean wheat grain yield (t/ha) for the same thirteen fungicide treatments, ranked from lowest to highest productivity.

The evaluation of 13 chemically active ingredients revealed significant variation in their efficacy against wheat stripe rust, as measured by disease severity and crop yield (Figure 4). Pyraclostrobin + Epoxyconazole demonstrated the most effective disease control with a mean severity of 0.18%, followed by Tebuconazole + Trifloxystrobin (0.42%) and Azoxystrobin (0.89%). In contrast, Propiconazole exhibited the highest disease severity at 26.25% (*n* = 22), indicating relatively poor disease suppression (Figure 4A). Regarding yield performance, Bixafen + Propiconazole emerged as the superior treatment, producing the highest mean yield of 6.42 t/ha based on the most robust dataset (*n* = 26). This fungicide combination maintained moderate disease control (13.37% severity) while maximizing productivity (Figure 4B). Tebuconazole ranked second with 5.59 t/ha (*n* = 11), demonstrating consistent performance with 24.53% mean severity. Conversely, Propiconazole showed the poorest yield performance at 2.96 t/ha, coupled with high disease severity, suggesting inadequate protection against stripe rust infection. These findings reveal an inverse relationship between disease severity and crop yield across most treatments. Notably, while some fungicides such as Pyraclostrobin + Epoxyconazole achieved excellent disease control, their yield benefits (3.22 t/ha) were limited compared to Bixafen + Propiconazole, which balanced both disease suppression and productivity optimization.

#### 2.1.3. Cultivar-Specific Responses to Chemical Control Against Stripe Rust

**Figure 5:**
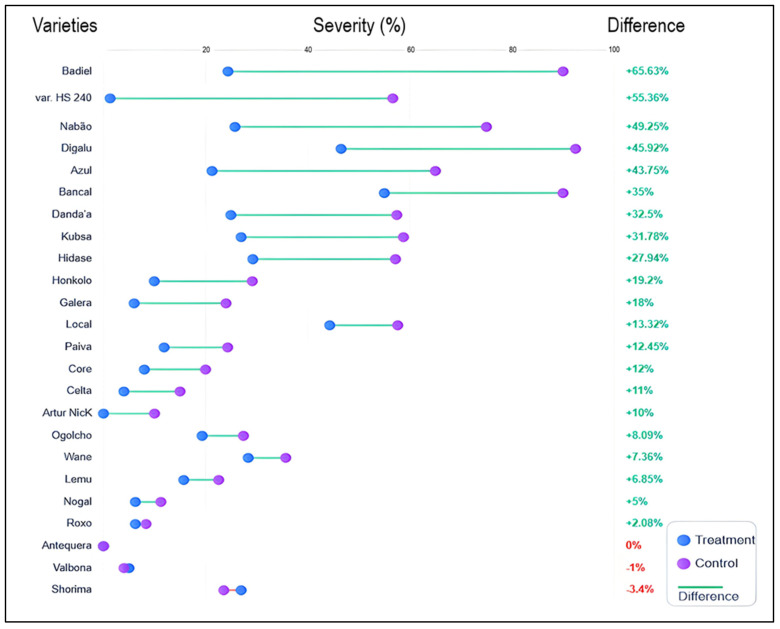
Cultivar-specific responses to chemical fungicide application, demonstrating differential susceptibility and treatment efficacy across 24 wheat varieties against stripe rust disease. The dumbbell plot displays paired comparisons of mean disease severity (%) (infected leaf surface) for each wheat variety under two conditions: fungicide-treated (blue circles) and untreated control (purple circles), connected by horizontal lines indicating the magnitude of treatment effect. Varieties are ranked from highest to lowest percent difference in disease severity reduction. The experiments were conducted in three replicates (*n* = 3).

Figure 5 demonstrates substantial variability in stripe rust severity across 24 wheat cultivars under both chemical treatment and control conditions. The cultivars exhibited a wide range of disease reduction responses, with percentage differences ranging from −3.4% (Shorima) to + 65.63% (Badjel), indicating that chemical control efficacy is strongly cultivar-dependent. Highly susceptible cultivars such as Badjel and var. HS 240 showed the greatest absolute severity reductions (approximately 55–65%), demonstrating that fungicides are most effective when baseline disease pressure is high. Moderately susceptible cultivars like Nabão, Digalu, Azul, and Bancal exhibited intermediate responses (27–49% reduction), while cultivars with inherent resistance, such as Wane, Lemu, Nogal, Roxo, Antequera, Valbona, and Shorima, showed minimal disease severity differences between treated and control plots (0–12% reduction). Notably, three cultivars (Antequera, Valbona, and Shorima) displayed negative or near-zero percentage differences, suggesting these genotypes possess sufficiently strong genetic resistance that chemical intervention provides negligible additional protection.

### 2.2. Biological Control

#### 2.2.1. Effect of Biological Control on Stripe Rust Disease Severity and Yield

**Figure 6:**
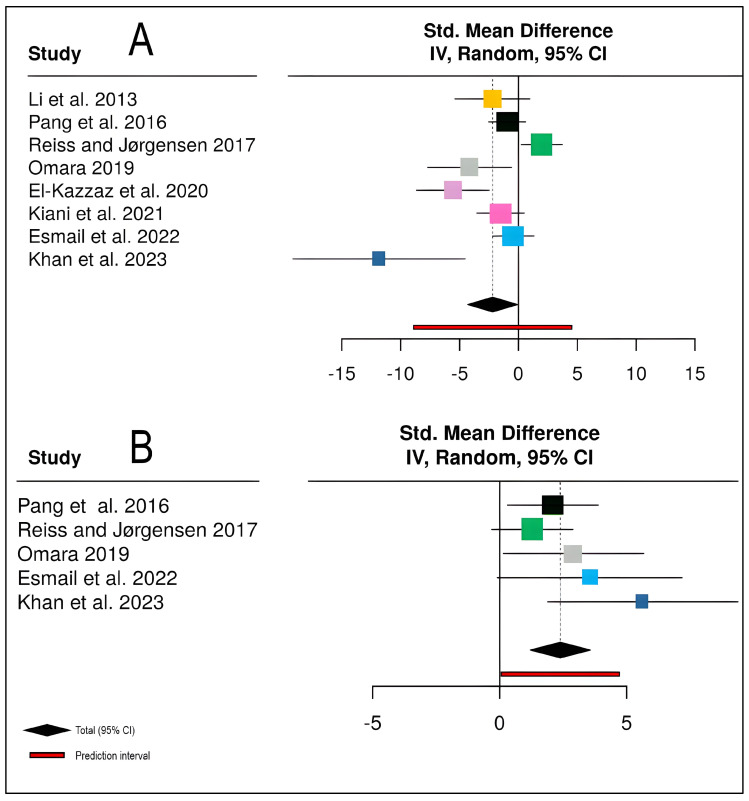
Meta-analysis forest plots demonstrating the effectiveness of biological control interventions on stripe rust disease management and wheat productivity: (**A**) Standardized mean difference (SMD) for stripe rust disease severity across eight studies (*n* = 8) [18,19,20,21,22,23,24,31], showing that chemical control significantly reduces disease severity (negative SMD values indicate disease reduction). (**B**) Standardized mean difference for wheat yield across the five studies (*n* = 5) [19,21,22,23], demonstrating that chemical control significantly increases yield (positive SMD values indicate yield improvement). Effect sizes were pooled using a random-effects model with inverse variance (IV) weighting. Each square represents the point estimate of individual study effects, with the square size proportional to study weight in the meta-analysis. Horizontal lines represent 95% confidence intervals (CI) for each study. The black diamond at the bottom indicates the pooled effect estimate with its 95% CI, while the red bar represents the prediction interval for future studies. The vertical dashed line at zero represents no effect.

The meta-analysis of biological control effects on stripe rust severity encompassed eight studies [18,19,20,21,22,23,24,31] with a total of 57 participants in experimental groups and 16 in control groups (Figure 6). The pooled analysis demonstrated that biological control agents significantly reduced disease severity compared to untreated controls, with a standardized mean difference of −2.19 (95% CI: −4.29 to −0.10, *Z* = −2.05, *p* = 0.0405). However, individual study effect sizes varied considerably, ranging from −11.83 to 1.98, with study weights between 5.5% and 15.1%. The analysis revealed substantial heterogeneity among studies (*I*^2^ = 78.5%, *τ*^2^ = 6.9307, *χ*^2^ = 32.57, *df* = 7, *p* < 0.0001), indicating that approximately 79% of the observed variance reflects true differences in treatment effects rather than sampling error. This high heterogeneity suggests considerable variability in biocontrol efficacy across different studies, potentially attributable to variations in biocontrol agent types, application methods, environmental conditions, or pathogen strains (Figure 6A).

For yield outcomes, five studies [19,21,22,23] contributed data from 39 experimental and 10 control participants (Figure 6B). Biological control demonstrated a highly significant positive effect on yield, with a pooled SMD of 2.39 (95% CI: 1.21 to 3.56, *Z* = 3.99, *p* < 0.0001), indicating substantial yield improvements in biocontrol-treated plots. All individual studies showed positive effect estimates with study weights ranging from 9.0% to 35.5%, and confidence intervals for all studies excluded zero. The analysis exhibited low to moderate heterogeneity (*I*^2^ = 24.2%, *τ*^2^ = 0.3442, *χ*^2^ = 5.28, *df* = 4, *p* = 0.2597), suggesting relatively consistent yield responses across different study contexts despite variations in biocontrol agents and experimental conditions. The prediction interval [0.06; 4.71] indicates that future studies would consistently demonstrate positive yield effects, with most falling well above the null value. These findings collectively demonstrate that biological control agents, including bacterial strains such as *Bacillus subtilis* and *Pseudomonas putida*, effectively suppress stripe rust while enhancing wheat yields. The substantially higher heterogeneity in disease severity responses compared to yield outcomes suggests that while biocontrol efficacy against the pathogen may vary considerably depending on specific implementation factors, the ultimate benefit to crop productivity remains robust and consistent across diverse conditions.

#### 2.2.2. Publication Bias Assessment for Biological Control Studies

**Figure 7:**
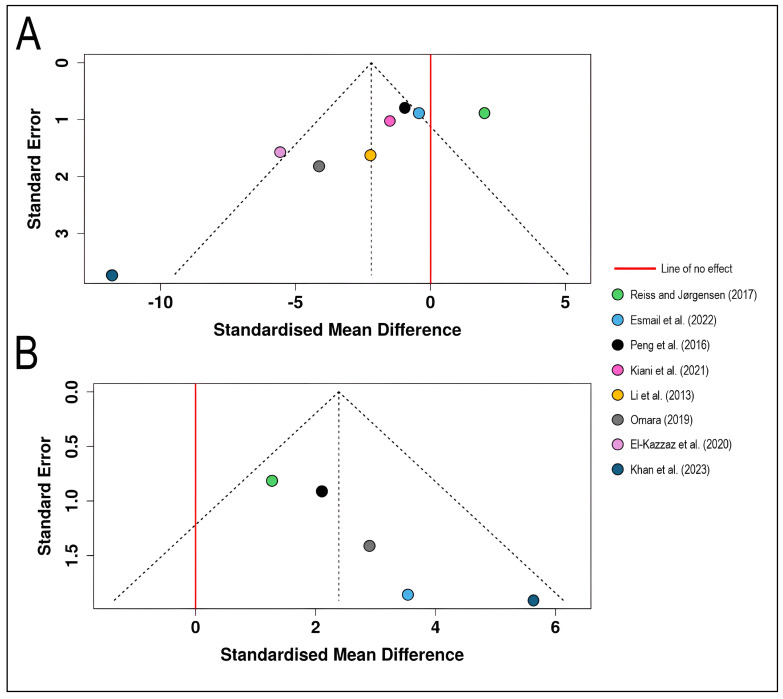
Funnel plots for visual assessment of publication bias in the meta-analysis of biological control effects on wheat stripe rust: (**A**) Funnel plot for disease severity outcomes (*n* = 8 studies) [18,19,20,21,22,23,24,31] showing the relationship between standardized mean difference (*x*-axis) and standard error (*y*-axis, inverted scale with larger, more precise studies at the top). (**B**) Funnel plot for wheat yield outcomes (*n* = 5 studies) [19,21,22,23]. Each colored circle represents an individual study, positioned according to its effect size and precision. The dashed diagonal lines form a pseudo-confidence region representing the expected 95% distribution of studies in the absence of heterogeneity and bias. The solid red vertical line indicates the line of no effect (SMD = 0).

The funnel plot for disease severity outcomes displays standardized mean differences from eight studies [18,19,20,21,22,23,24,31] plotted against their standard errors (Figure 7). The plot exhibits pronounced visual asymmetry, with studies scattered irregularly across a wide range of effect sizes spanning from approximately −15 to + 5 on the standardized mean difference scale. A notable outlier, ref. [19] appears far outside the funnel boundaries on the left side with exceptionally high standard error (approximately 3.7) and extreme negative effect size (SMD ≈ −11.83), suggesting substantial disease reduction but with considerable uncertainty. Most studies cluster in the moderate precision range (standard errors between 1.0 and 2.0), with several positioned to the left of the pooled effect estimate, while one study [21] shows a positive effect estimate on the right side of the no-effect line. This asymmetry and heterogeneous distribution may reflect several factors beyond publication bias, including genuine variation in biocontrol efficacy across different agents, application protocols, environmental conditions, or pathogen pressure, which aligns with the high statistical heterogeneity (*I*^2^ = 78.5%) observed in the corresponding forest plot analysis. The funnel plot indicates a potential publication bias. The Egger’s test supports the presence of funnel plot asymmetry (*intercept* = −4.4, 95% CI = −6.98–−1.82, *t* = −3.344, *p* = 0.016) (Figure 7A).

The funnel plot for yield outcomes presents five studies [19,21,22,23] with all effect estimates positioned to the right of the line of no effect, indicating consistent positive yield responses to biological control interventions. The studies demonstrate a more balanced distribution across precision levels, with standard errors ranging from approximately 0.2 to 2.0. The plot shows relatively greater symmetry compared to the severity analysis, with studies distributed fairly evenly around the central vertical axis and mostly contained within the expected funnel boundaries. Ref. [19] study again exhibits the largest effect size (SMD ≈ 5.64) with relatively high standard error (approximately 2.0), while studies with higher precision (lower standard errors) cluster around more moderate effect estimates (SMD ≈ 2–3). This pattern is consistent with the low-to-moderate heterogeneity (*I*^2^ = 24.2%) observed in the yield meta-analysis, suggesting more uniform treatment benefits across studies despite variations in biocontrol agents and experimental conditions. The funnel plot indicates a potential publication bias. The Egger’s test supports the presence of funnel plot asymmetry (*intercept* = 2.85, 95% CI = 1.52–4.17, *t* = 4.207, *p* = 0.025) (Figure 7B).

**Figure 8:**
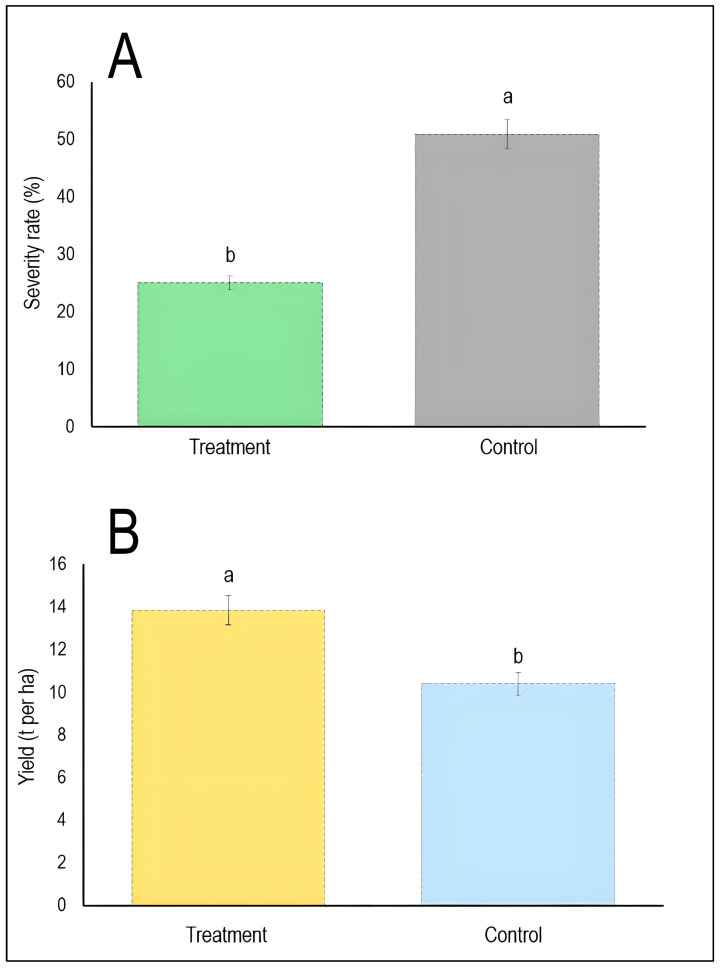
Comparative efficacy of biological control on stripe rust severity (**A**) and wheat yield (**B**). Bars represent mean values pooled across all included studies (*n* = 8 for severity, and *n* = 5 for yield) [18,19,20,21,22,23,24,31], with vertical error bars indicating standard deviation. Different lowercase letters (a, b) above bars denote statistically significant differences between treatment and control groups (*p* < 0.05) according to the Tukey HSD test. The dashed horizontal line at the top of each bar represents the upper limit of the standard deviation.

Regarding treatment-control comparisons (Figure 8), it has been shown that biological control treatments significantly reduced stripe rust severity to approximately 25% compared to 51% in untreated controls. This represents roughly 50% disease suppression (Figure 8A). Furthermore, the biologically treated plots achieved mean yields of approximately 13.8 t/ha compared to 10.5 t/ha in controls, representing a 31% yield increase. However, the difference may not reach full statistical significance at the conventional *p* < 0.05 level (Figure 8B). This suggests that biological control agents effectively suppress disease severity with statistically significant results, though their impact on yield enhancement may be more variable.

#### 2.2.3. Strain-Specific Efficacy of Biological Control Agents Against Stripe Rust

**Figure 9:**
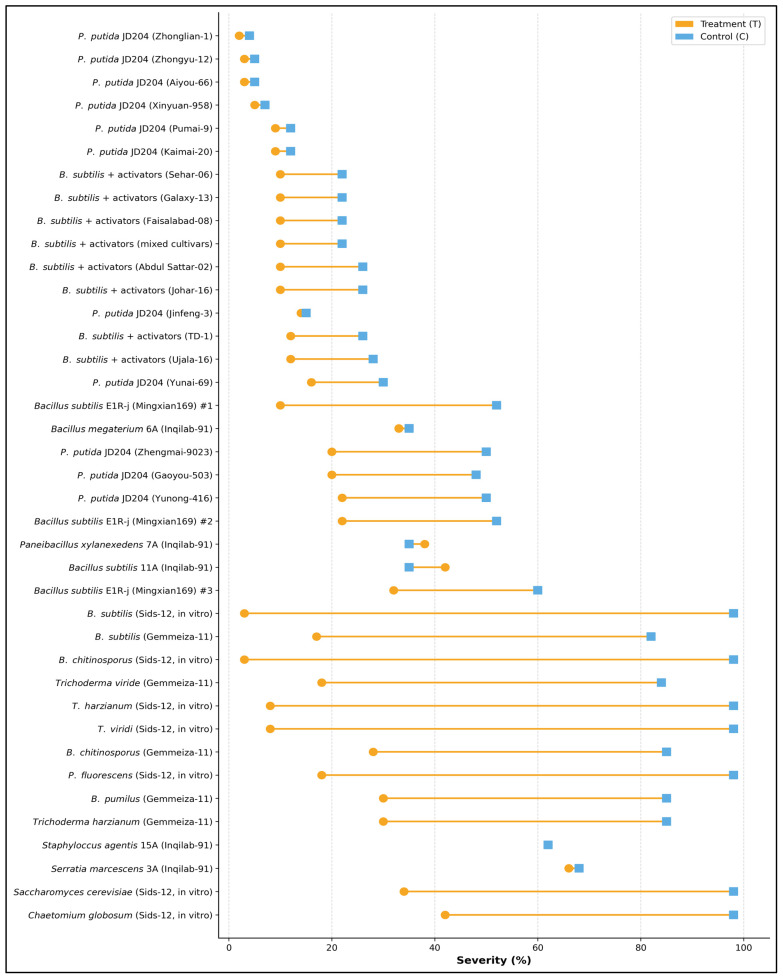
Strain-cultivar specific efficacy of biological control agents (BCAs) demonstrating differential disease suppression across 35 combinations of microbial antagonists and wheat varieties against stripe rust. The dumbbell plot compares mean stripe rust disease severity (%) (infected leaf surface) between BCA-treated plots (orange circles) and untreated controls (blue squares), with horizontal lines connecting paired observations for each strain-cultivar combination. Combinations are ranked by treatment efficacy, from most effective disease reduction (top) to least effective or adverse responses (bottom), and experiments were conducted in three replicates (*n* = 3).

The substantial heterogeneity in biocontrol efficacy was highlighted across different microbial strains and species tested against stripe rust (Figure 9). Among *Pseudomonas putida* isolates (JD204 strains), performance varied markedly, with some strains achieving near-complete disease suppression (treatment severity < 10%) while others showed minimal effectiveness, with control severity levels remaining above 50%. The *Bacillus subtilis* formulations combined with activators exhibited moderate and relatively consistent disease control across multiple applications (Sehar-06, Galaxy-13, Faisalabad-08, mixed cultivars, Abdul Sattar-02, Johar-16, TD-1, and Ujala-16), reducing severity from approximately 25–30% in treated plots to 30–45% in controls. Notably, the most potent biocontrol agents were *Bacillus subtilis* E1Rj (Mingxian169) strains (#1, #2, and #3) and several in vitro tested antagonists (*B. subtilis* Sids-12, *B. chitinosporus* Sids-12, *Trichoderma* species, *T. harzianum* Sids-12, and *T. viridi* Sids-12), which maintained treatment severities below 30% while controls exceeded 80–95%, representing disease reductions exceeding 60–70%. Furthermore, species-specific differences in antagonistic capacity were evident, with *Serratia marcescens* 3A and *Staphylococcus agentis* 15A showing limited disease control (treatment severity > 60%), whereas *Bacillus megaterium* 6A, *Paenibacillus xylanexedens* 7A, and *B. subtilis* 11A demonstrated intermediate efficacy (treatment severity 35–50%). *Trichoderma* and *Bacillus* species formulated for in vitro conditions displayed superior performance compared to field-applied isolates, likely reflecting optimized culture conditions and higher cell densities (Figure 9).

#### 2.2.4. Protective and Curative Efficacy of Bacterial Biocontrol Agents Against Stripe Rust

**Figure 10:**
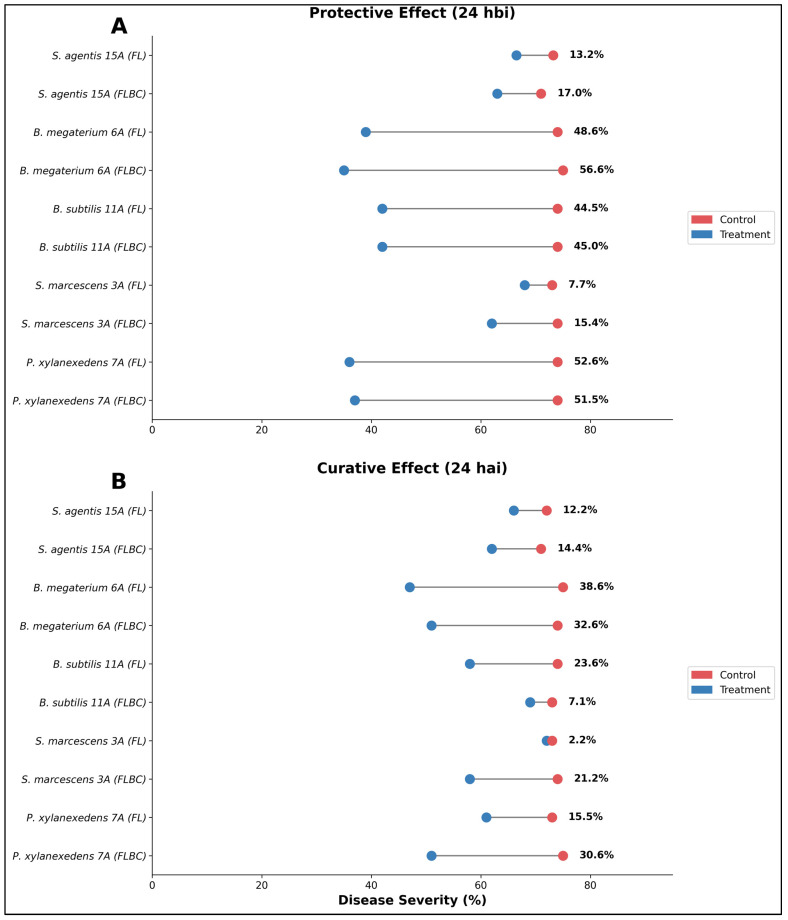
Comparative efficacy of bacterial biocontrol formulations applied as protective versus curative treatments against wheat stripe rust disease. Dumbbell plots show paired comparisons of mean disease severity (%) (infected leaf surface) between biocontrol-treated plots (blue circles) and untreated controls (red circles) at 24 h post-application, with horizontal lines connecting paired observations for each strain-formulation combination. The experiments were conducted in 7-day-old seedlings of the Inqilab-91 variety for each biocontrol formulation applied three times (*n* = 3) as fermented liquid (FL) and fermented liquid with bacterial cells (FLBC). (**A**) Protective effect: Biological control agents were applied 24 h before inoculation (24 hbi) to evaluate preventive activity against pathogen establishment. (**B**) Curative effect: Biological control agents were applied 24 h after inoculation (24 hai) to assess post-infection therapeutic activity and ability to halt disease progression.

Kiani et al. [31] illustrated the protective effect of five endophytic bacterial strains applied 24 h before *Puccinia striiformis* inoculation (24 hbi) using two formulation types: fermented liquid (FL) and fermented liquid with bacterial cells (FLBC) (Figure 10). *Bacillus megaterium* 6A demonstrated the highest protective efficacy, achieving 56.6% (FLBC) and 48.6% (FL) disease reduction, followed by *Paenibacillus xylanexedens* 7A with 51.5% (FLBC) and 52.6% (FL) reduction. *Bacillus subtilis* 11A showed moderate protective capacity with 45.0% (FLBC) and 44.5% (FL) disease suppression, while *Serratia marcescens* 3A exhibited variable performance between formulations (15.4% FLBC vs. 7.7% FL). *Staphyloccus agentis* 15A displayed the weakest protective effect, achieving only 17.0% (FLBC) and 13.2% (FL) reduction (Figure 10A).

Figure 10B presents the curative effect when formulations were applied 24 h after inoculation (24 hai), revealing substantially different efficacy patterns compared to protective applications. *Bacillus megaterium* 6A maintained relatively strong curative activity with 32.6% (FLBC) and 38.6% (FL) disease reduction, though notably lower than its protective performance. *Bacillus subtilis* 11A showed markedly reduced curative efficacy (7.1% FLBC vs. 23.6% FL), indicating that cell-free filtrates may contain more stable antimicrobial compounds effective in post-infection scenarios. *Paenibacillus xylanexedens* 7A demonstrated moderate curative capacity (30.6% FLBC, 15.5% FL), while *Serratia marcescens* 3A exhibited minimal curative activity (2.2% FL), with FLBC formulation performing better (21.2%). *Staphyloccus agentis* 15A maintained consistent low efficacy in both modes (12.2% FL, 14.4% FLBC).

### 2.3. Comparative Efficacy Analysis for Stripe Rust Management

**Figure 11:**
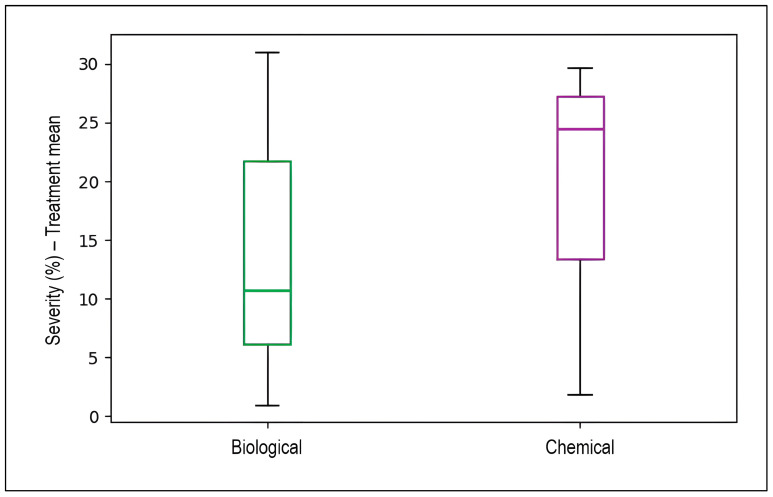
Comparative efficacy of biological versus chemical control strategies for managing wheat stripe rust disease severity. Box plots display the distribution of mean treatment efficacy (measured as disease severity percentage or infected leaf surface in treated plots) across all experiments evaluating each control approach (*n* = 72). Each box represents the interquartile range (IQR) containing the middle 50% of observations, with the horizontal line inside each box indicating the median value. Vertical whiskers extend to the minimum and maximum values within 1.5 × IQR from the box edges, representing the range of typical observations.

Figure 11 presents box plots comparing disease severity reduction (treatment mean minus control) between biological and chemical control strategies. Chemical control demonstrated a higher median severity reduction (approximately 24%) with values ranging from approximately 13% to 30%. In contrast, biological control exhibited lower median severity reduction (approximately 10.5%) with substantially greater variability, as evidenced by the wider interquartile range spanning from approximately 6% to 22% and extended whiskers reaching from approximately 1% to 31%. This greater variability in biological control reflects the strain-dependent and environmentally sensitive nature of microbial biocontrol agents, whose efficacy can fluctuate based on factors such as application timing, formulation quality, environmental conditions, and antagonistic strain characteristics. Chemical fungicides, by contrast, provide more consistent and predictable disease suppression due to their direct mode of action against the pathogen.

## 3. Discussion

This meta-analysis provides a comprehensive quantitative compilation of chemical and biological techniques for controlling stripe rust in wheat in different agroecological contexts. Taking into account 156 experimental comparisons from open-field and greenhouse studies, the analysis clarifies the scope, consistency, and variability of treatment impacts on disease severity and grain yield, while identifying cultivar preferences and implementation factors as key moderators.

Chemical fungicides consistently exhibited strong efficacy in reducing stripe rust severity across studies (SMD = −1.04) and increasing wheat productivity (SMD = 1.30), with low to moderate variability between studies. These data corroborate multiple field reports attesting to the remarkable effectiveness of triazoles, strobilurins, and SDHI-containing mixtures in combating *Puccinia striiformis* epidemics [12,14,15]. The lack of heterogeneity in yield response (*I*^2^ = 0%) indicates that, despite differences in fungicide formulations and environmental conditions, the chemical approach consistently results in disease reduction and improved productivity. However, specific studies on plant varieties have shown that the benefits of fungicides are not consistent. Highly susceptible varieties showed the most significant absolute reductions in disease severity and the most notable yield improvements, while resistant varieties showed a negligible additional benefit from chemical action. This model illustrates the significant influence of host genetic resistance on treatment response and aligns with previous studies suggesting that fungicides are more effective when initial resistance is low and disease pressure is high [6,9]. These findings highlight the need to consider variety resistance profiles when selecting fungicides in order to maximize input efficiency and prevent unnecessary treatments. Although chemical fungicides are effective, their use raises questions about sustainability. Frequent use imposes strong selection pressure on pathogen populations, accelerating the emergence of resistant strains, especially against QoI and DMI fungicides [16,17]. Environmental contamination and adverse impacts on non-target microbial communities intensify this risk, highlighting the limitations of long-term chemical disease management.

Biological agents significantly reduced the severity of stripe rust (SMD = −2.19) and improved wheat productivity (SMD = 2.39), proving that microbial antagonists can produce effects similar to those of chemical fungicides. However, the elimination of the disease revealed significant heterogeneity (*I*^2^ = 78.5%), indicating considerable disparity between studies. This variability is biologically credible and reflects variations between antagonist species, strain identity, composition, timing of application, environmental conditions, and varietal context [21,32,33]. In contrast to the results concerning severity, responses related to the effectiveness of biological control were fairly stable (*I*^2^ = 24.2%), indicating that microbial agents are capable of increasing productivity through mechanisms that go beyond the direct eradication of pathogens. These processes likely include induced systemic resistance, optimization of nutrient assimilation, regulation of phytohormones, and improvement of plant physiological resilience [19,22,25]. The independence between fluctuations in disease eradication and consistent yields suggests that biological control can provide agronomic benefits even when disease control is only partial. Furthermore, strain-level studies have revealed that not all biological control agents are created equal. *Bacillus subtilis*, *B. megaterium*, *Paenibacillus xylanexedens*, and *Trichoderma* spp. consistently outperformed other taxa, while some bacterial strains demonstrated limited or variable efficacy. These findings highlight that the success of biological control is primarily strain-specific and cannot be applied uniformly to the entire genus, emphasizing the importance of careful strain selection and formulation refinement [23,24].

In this study, chemical control reduced stripe rust severity with an SMD of −1.04, representing a large effect size and a substantial decrease in disease severity under several conditions, especially in the field. Similarly, biological control interventions produced an even larger reduction in disease severity SMD = −2.19, indicating a very strong suppressive effect relative to untreated controls. Such magnitudes of effect are agronomically meaningful and comparable to disease suppression levels required for effective stripe rust management in commercial wheat production systems. However, the higher heterogeneity associated with biological control outcomes highlights the strain- and context-dependent nature of microbial efficacy, emphasizing the importance of careful agent selection and optimized field application strategies.

In chemical and biological approaches, variety identity has been shown to be a crucial factor in treatment effectiveness. Variations in responses illustrate the genetic diversity associated with stripe rust resistance, including strain-specific Yr genes and adult plant resistance processes [1,32]. Resistant varieties showed minimal infection rates and marginal improvements with external inputs, while susceptible varieties showed strong responsiveness to both fungicides and biological control agents. This dependence on cultivated varieties offers a mechanistic explanation for a significant portion of the disparity observed in the meta-analysis, while emphasizing the importance of incorporating host genetics into disease management models. Ignoring variety resistance could lead to an overestimation of treatment impact and suboptimal advice. Rather than viewing chemical and biological control as competing options, the findings argue for a complementary role within integrated disease management (IDM) systems.

The use of chemical fungicides remains essential for the rapid elimination of epidemics, especially in the case of highly susceptible cultivars exposed to high pathogen pressure. Despite their variability in disease eradication, biological control agents offer lasting benefits in terms of productivity and are consistent with agroecological goals as they reduce the use of chemicals, promote microbial diversity, and limit the development of resistance [26]. The strategic combination of resistant varieties, targeted use of fungicides, and well-identified biological control agents could optimize disease management while minimizing environmental impact. The preventive use of biological control, followed by a reduction in fungicide doses, is a promising approach for the sustainable control of stripe rust, especially with the potential increase in disease risk associated with climate change [11].

The findings of this meta-analysis indicate that chemical and biological control strategies may play complementary roles in the management of wheat stripe rust. Chemical fungicides exhibited more consistent and stable effects across studies, reflecting their rapid and direct suppression of pathogen development, whereas biological control agents showed larger but more variable effects, likely due to differences in microbial strains, application timing, formulation, and environmental conditions. Similar patterns have been widely reported in the plant disease management literature, where chemical control provides reliable short-term disease suppression, while biological control contributes to longer-term disease regulation and sustainability within integrated pest management frameworks.

Recent research has increasingly emphasized the potential benefits of integrating biological control agents with reduced fungicide inputs, reporting effective disease suppression alongside decreased chemical use, lower selection pressure for fungicide resistance, and improved environmental compatibility [29,33]. Although the present meta-analysis did not directly evaluate combined chemical–biological treatments due to limited availability of suitable comparative data, the observed consistency of fungicide effects and the strong, albeit variable, performance of biological agents support the rationale for integrated approaches. These findings highlight the need for future field studies specifically designed to quantify the combined effects of chemical and biological control strategies on stripe rust severity and wheat yield.

## 4. Materials and Methods

### 4.1. Data Selection

This study aimed to quantitatively compare the effectiveness of chemical fungicides and biological control agents in reducing stripe rust severity and improving wheat yield across diverse agroecological conditions using a meta-analytical framework. The objective was also to provide an overview of the methodological factors that may determine the extent of the effects of antagonists on leaf stripe rust and yield. Finally, we propose a new theoretical framework focused on the interaction of antagonists with the stripe rust pathogen, types of application and treatment, and the scale of the study as potential parameters determining the impacts of biocontrol and chemical control on disease severity and wheat production. The data used for the meta-analysis were extracted from the literature published in peer-reviewed journals over two decades (2005–2025) through searches on Google Scholar using the PRISMA model (see Supplementary Figure S1 and Table S1). The following keywords were used: “Puccinia striiformis” OR “Stripe rust” AND “Wheat” OR “Triticum aestivum” AND “Chemical control” OR “Fungicide” AND “biological control” OR biocontrol OR “biofungicide”. Among 172 articles on the control of stripe rust in wheat using chemical fungicides and microorganisms (bacteria and fungi) as antagonistic species, only 12 articles provided combinations and results in common to perform a rigorous and relevant meta-analysis to address the difference between these two types of treatments, given that the use of fungicides has harmful effects on the environment and also on human health.

Meta-analyses were conducted separately for chemical and biological control interventions to assess their effects on stripe rust disease severity and wheat yield.

Disease severity (%) was calculated as the proportion of infected leaf area relative to total leaf area using the modified Cobb scale [34], and expressed with this formula:

$$\mathrm{Severity}\;\mathrm{rate}\;(\%)\;=\;\frac{\sum (n_{i}\times v_{i})}{N\times V}\times 100$$

where *n*_i_ is the number of leaves in each severity class, *v*_i_ is the corresponding severity score, *N* is the total number of assessed leaves, and *V* is the maximum severity score.

Grain yield was determined at harvest maturity following standard agronomic procedures described in wheat field trials [35,36]. Briefly, wheat plants were harvested from each experimental plot, threshed, and cleaned, and the total grain weight was recorded. Yield was expressed on an area basis, most commonly as tons per hectare (t ha^−1^), using the following calculation:

$$\mathrm{Grain}\;\mathrm{yield}\;(t\;{\mathrm{ha}}^{-1})\;=\;\frac{Grain\;weight\;(kg)}{Harvested\;area\;(m^{2})}\times 10$$

When reported in the original studies, grain yield values were adjusted to a standard grain moisture content (typically 12–14%) prior to analysis, in accordance with established agronomic conventions. For the meta-analysis, yield data were extracted directly from the published literature and used to calculate standardized mean differences comparing treated and untreated control groups.

### 4.2. Statistical Analysis

Prior to conducting meta-analyses, data normality was assessed using Shapiro–Wilk tests for continuous variables. Standardized mean differences (SMD) were calculated as the primary effect size measure to account for heterogeneity in measurement scales across studies. SMD values of 0.2, 0.5, and ≥0.8 were interpreted as small, moderate, and large effects, respectively [37]. Negative SMD values indicate disease reduction relative to untreated controls, whereas positive values indicate yield improvement.

Random-effects models using the inverse variance (IV) method were employed for all meta-analyses, acknowledging anticipated variability in treatment effects attributable to differences in biocontrol agents, fungicide formulations, wheat cultivars, environmental conditions, and experimental designs. Between-study heterogeneity was quantified using Cochran’s Q test (*χ*^2^), the between-study variance (*τ*^2^), and the inconsistency statistic (*I*^2^), which represents the percentage of total variation attributable to heterogeneity rather than sampling error. *I*^2^ values of 25%, 50%, and 75% were interpreted as indicating low, moderate, and high heterogeneity, respectively [38]. Overall treatment effects were evaluated using Z-tests, with statistical significance set at *α* = 0.05. Forest plots were constructed to visualize individual study effects, pooled estimates with 95% confidence intervals (CI), and study weights. Prediction intervals were calculated to estimate the range of true effects expected in future studies, accounting for between-study heterogeneity.

Publication bias was assessed through visual inspection of funnel plots, wherein study effect sizes were plotted against their standard errors. Symmetrical funnel distribution around the pooled effect estimate indicates the absence of publication bias, while asymmetry may suggest selective reporting of statistically significant results or small-study effects. For comparative analyses of treatment versus control groups, one-way analysis of variance (ANOVA) was performed, followed by post hoc pairwise comparison tests. Statistical significance groups were denoted using Tukey’s Honestly Significant Difference (HSD) Test, where treatments sharing common letters indicate non-significant differences at *p* < 0.05.

All statistical analyses were performed using Python (version 3.11) with specialized packages including PythonMeta (version 1.26) for meta-analytic calculations, matplotlib (version 3.10.8) and seaborn (version 0.13.2) for data visualization, and scipy (version 1.16.3) and statsmodels (version 0.14.6) for additional statistical procedures. Statistical significance was consistently evaluated at the *p* < 0.05 level for all hypothesis tests, and 95% confidence intervals were reported for all effect size estimates.

## 5. Conclusions

This meta-analysis provides the first comprehensive quantitative assessment of chemical and biological control methods for stripe rust in wheat in various agroecological environments worldwide. The use of chemical fungicides consistently leads to a significant reduction in disease and improved yields, demonstrating their short-term effectiveness. Despite greater variability in disease control, biological agents provide significant benefits in terms of productivity and offer significant advantages in terms of the environment and resistance management. The conclusions indicate that biological control is not a substitute for fungicide use during severe epidemics, but is an effective additional tool in the context of integrated disease management systems. Future research should focus on validation in real-world conditions at various sites, refinement of strain formulation, and the combination of biological control agents with host resistance and reduced fungicide treatments. Integrated methods such as these are essential to ensure robust, profitable, and environmentally friendly wheat production in response to the evolving risks associated with stripe rust and climate change.

## Figures and Tables

**Table 1:** Fungicide product information and application timing for uniform fungicide application timing conducted between 2005 and 2025 under field conditions to manage stripe rust.

Fungicide Active Ingredient	Target Site of Action	Fungicides Classes	Fungicide Resistance Action Committee (FRAC) Code	Application Spray Times	Fungicide Application Timing	References
Bixafen + Propiconazole	C2 + G1	SDHI + DMI (SBIs)	7 + 3	1	GS34 (jointing)	[12]
				2	GS34 (jointing) and GS44 (booting)	
Tebuconazole	G1	DMI (SBIs)	3	1	Growth stage	[13]
				2	Appearance of disease	[30]
Propiconazole	G1	DMI (SBIs)	3	1	Growth stage, booting, heading	[13]
				2	Appearance of disease, booting, heading, Tillering-Booting	[30]
				3	Booting, heading	[15]
Tebuconazole + Trifloxystrobin	G1 + C3	DMI + QoI	3 + 11	1	Tillering-Booting	[14]
				2	Appearance of disease	[30]
Azoxystrobin + Difenconazole	C3 + G1	QoI + DMI	11 + 3	2	Appearance of disease	[30]
Azoxystrobin	C3	QoI	11			
Tricyclazole (triazolobenzo- thiazole) + Mancozeb (dithio-carbamates)	I1 + Multi-site contact activity	MBI-R + Dithiocarbamates and relatives (electrophiles)	16.1 + M 03			
Hexaconazole (triazoles) + Zineb (Zinc ethylenebis-(dithiocarbamate)	G1 + Multi-site contact activity	DMI + Dithiocarbamates and relatives (electrophiles)	3 + M 03			
Pyraclostrobin (methoxy-carbamates) + Epoxiconazole	C3 + G1	QoI + DMI	11 + 3			
Hexaconazole + Captan (phthalimides)	G1 + Multi-site contact activity	DMI + Phthalimides (electrophiles)	3 + M 04			
Kresoxim-methyl (oximino-acetates)	C3	QoI	11			
Difenoconazole	G1	DMI	3			

QoI: Quinone outside inhibiting fungicide class; SDHI: Succinate dehydrogenase inhibiting fungicide class; DMI: Demethylation inhibiting fungicide class; SBIs: Sterol Biosynthesis Inhibitors; C2: Complex II; succinate-dehydrogenase; C3: complex III: cytochrome bc1 (ubiquinol oxidase) at Qo site (cyt b gene); G1: C14-demethylase in sterol biosynthesis (erg11/cyp51); I1: Reductase in Melanin Biosynthesis; MBI-R: Melanin Biosynthesis Inhibitors—Reductase.

## Data Availability

The data used for the analyses in this study are available within the article.

## Supplementary Materials

The following supporting information can be downloaded at https://www.mdpi.com/article/10.3390/plants15030412/s1, Figure S1: PRISMA flow diagram of global literature selection for the meta-analysis of chemical and biological control of wheat stripe rust; Table S1: Description of chemical and biological control studies included in the meta-analysis of wheat stripe rust management.

## Abbreviations

The following abbreviations are used in this manuscript:

ANOVA	Analysis of variance
CI	Confidence interval
DMI	Demethylation-inhibiting fungicide class
FAO	Food and Agriculture Organization
FL	Fermented liquid
FLBC	Fermented liquid with bacterial cells
FRAC	Fungicide Resistance Action Committee
GS	Growth stage
IDM	Integrated disease management
ISR	Induced systemic resistance
IV	Inverse variance
MBI-R	Melanin Biosynthesis Inhibitors—Reductase
ONSSA	Office National de Sécurité Sanitaire des Produits Alimentaires
PAL	Phenylalanine Ammonia-Lyase
POD	Peroxidase
PPO	Polyphenol Oxidase
PR	Pathogenesis-Related
PRISMA	Preferred Reporting Items for Systematic Reviews and Meta-Analyses
Pst	Puccinia striiformis f. sp. tritici
QoI	Quinone outside-inhibiting fungicide class
SAR	Systemic acquired resistance
SBIs	Sterol Biosynthesis Inhibitors
SDHI	Succinate dehydrogenase-inhibiting fungicide class
SMD	Standardized mean difference
SOD	Superoxide Dismutase
Yr	Yellow rust
hai	Hours after inoculation
hbi	Hours before inoculation
t/ha	Tons per hectare
