# Supplementary material for: Comparative Meta-Analysis of Chemical and Biological Strategies for the Management of Wheat Stripe Rust (Puccinia striiformis f. sp. tritici) Under Global Agro-Ecological Conditions
Source: Plants (Basel). 2026 Jan 29;15(3):412. doi: 10.3390/plants15030412 (PMC12899063; doi:10.3390/plants15030412)
Supplement: Supplementary file 1 [file plants-15-00412-s001.zip › plants-4105099-supplementary.pdf]

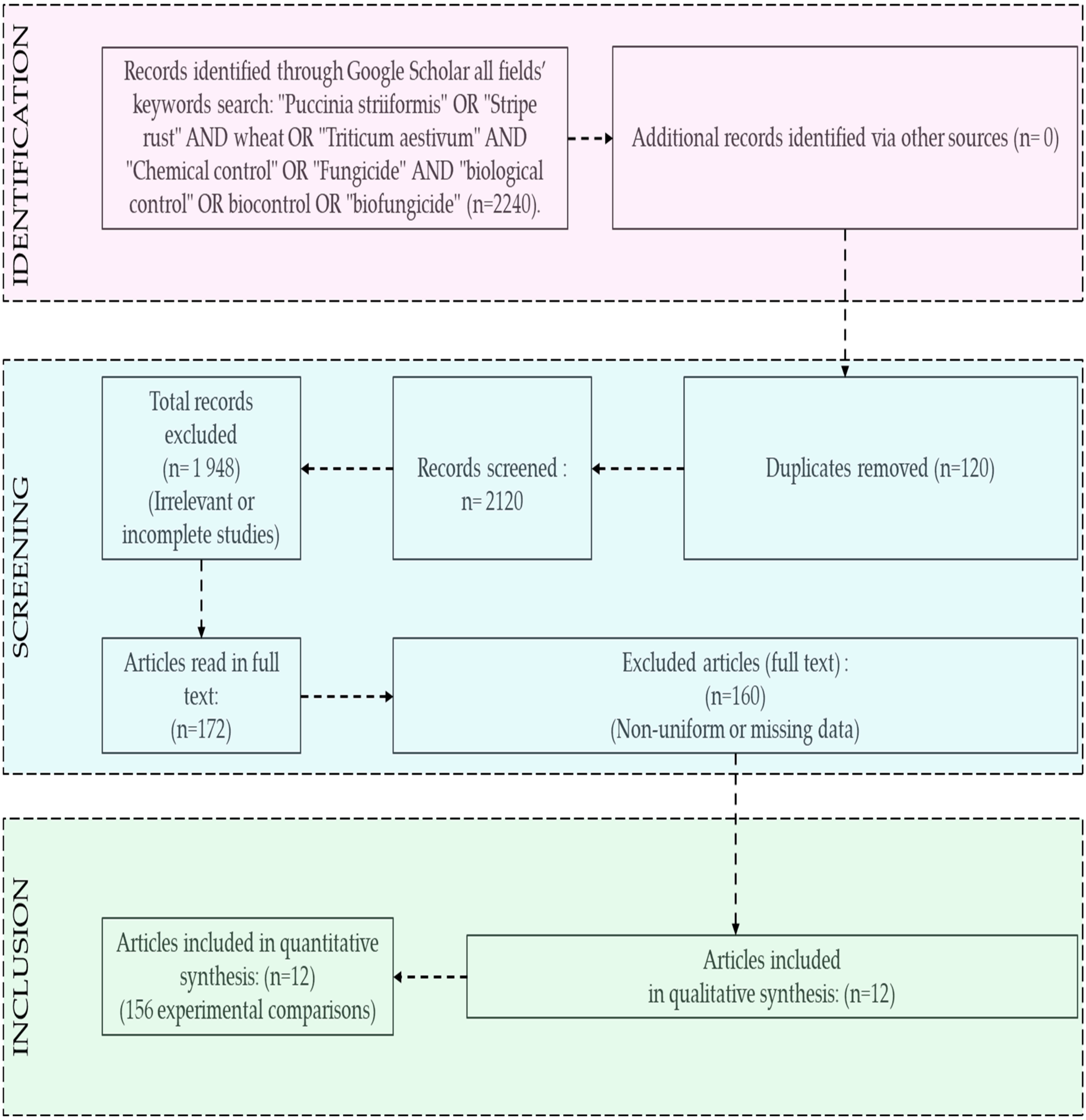

Figure S1. PRISMA flow diagram of global literature selection for the meta-analysis of chemical and biological control of wheat stripe rust.

Table S1. Description of chemical and biological control studies included in the meta-analysis of wheat stripe rust management.

Author	Year	Country	Control strategy	Active ingredient / Biocontrol agent	Mode of action	Wheat cultivar	Experimental conditions	References
Gomes et al.	2018	Portugal	Chemical	Bixafen + Propiconazole	SDHI+ DMI	13 cultivars	Field	[12]
Mengesha	2020	Ethiopia	Chemical	Tebuconazole, Propiconazole	DMI	5 cultivars	Field	[13]
Kebede et al.	2023	Ethiopia	Chemical	Tebuconazole + Trifloxystrobin	DMI+ QoI	4 cultivars	Field	[14]
Belay & Jenber	2024	Ethiopia	Chemical	Propiconazole	DMI	4 cultivars	Field	[15]
Li et al.	2013	China	Biological	*Bacillus subtilis* E1R-j	Antibiosis, competition for nutrients and space, and ISR	Mingxian 169	Greenhouse / Field	[18]
Pang et al.	2016	China	Biological	*Pseudomonas putida* JD204	Colonization, Competition, Antibiosis, and Induction of host defense mechanisms	11 cultivars	Greenhouse	[23]
Reiss & Jørgensen	2017	Denmark	Biological	*Bacillus subtilis* QST713	Antibiosis, competition, and ISR	Trappe	Field	[21]

Omara et al.	2019	Egypt	Biological	*Bacillus subtilis*<br>*Bacillus chitinospours*<br>*Bacillus pumilus*<br>*Trichoderma viride*<br>*Trichoderma harzianum*	Antibiosis, Mycoparasitism, Competition, and ISR	Gemmeiza-11	Greenhouse	[22]
El-Kazzaz et al.	2020	Egypt	Biological	*Trichoderma harzianum*<br>*Bacillus subtilis*<br>*Bacillus chitinosporus*	Antibiosis, Mycoparasitism, Competition, and ISR	Sids-12	Greenhouse/ Field	[20]
Kiani et al.	2021	Pakistan	Biological	*Paneibacillus xylanexedens* 7A<br>*Serratia marcescens* 3A<br>*Bacillus subtilis* 11A<br>*Bacillus megaterium* 6A<br>*Staphyloccus agentis* 15A	Antioxidant enzymes: SOD, POD, PPO, and PAL;<br>PR proteins	Inqilab-91	Semi-Field	[31]
Esmail et al.	2022	Egypt	Biological	*Trichoderma asperellum*<br>*Penicillium simplicissimum*	Mycoparasitism,<br>Cell wall–degrading enzymes,<br>Competition for nutrients, and<br>ISR	Sids-12	Greenhouse	[24]
Khan et al.	2023	Pakistan	Biological	*Bacillus subtilis* + elicitors (dextrose, chitosan, salicylic acid)	Antibiosis, and ISR	7 cultivars	Field	[19]
